# Supplementary material for: The impact of chewing khat during pregnancy on selected pregnancy outcomes in eastern Ethiopia: A cohort study with a generalized structural equation modeling analysis approach
Source: PLoS One. 2024 Aug 9;19(8):e0308681. doi: 10.1371/journal.pone.0308681 (PMC11315279; doi:10.1371/journal.pone.0308681)
Supplement: S1 Data — (DOCX) [file pone.0308681.s001.docx]

Questionnaire on assessment of effects of khat chewing during pregnancy on selected pregnancy outcomes in eastern Ethiopia.

Participant ANC follow up card ID___________

Date of Assessment/Extraction_______________________

**Participant/background characteristics at enrollment during** ANC

| **Part I: Sociodemographic and economic characteristics of participants** | | | |
| --- | --- | --- | --- |
| **No** | **Variables** | Coding category | Remark |
| 101 | Age | ----------------------in years |  |
| 102 | Are of residence | 1. Urban  2. Rural |  |
| 103 | What is your ethnicity? | 1. Oromo  2. Harari  3. Somali  4. Amhara  5. Others, specify_____________ |  |
| 104 | What is your religion? | 1. Muslim  2. Orthodox  3. Protestant  4. Catholic  5. Others, Specify…………… |  |
| 105 | Educational status  (What is the highest level of schooling attained) | 1. No formal education 2. Primary education (grade 1-8) 3. Secondary education (grade 9-12) 4. College/University completed 5. Post-graduate degree |  |
| 106 | Occupation status | 1. House wife/ Homemaker 2. Farmer 3. Government employee 4. Non-government employee 5. Self-employed/ Merchant 6. Student 7. Daily laborer 8. Unemployed 9. Other specify |  |
| 107 | Marital status | 1. Never married/single 2. Currently married 3. Divorced/separated 4. Widowed |  |
| 108 | Monthly income of the HH? | ______________________________ |  |
| Part II: **Substance use status during current pregnancy** | | | |
| 201 | Have you ever used any kind of substance during current pregnancy? | 1.Yes 2. No |  |
| 202 | Which kind of substance? | 1. Khat  2. Alcohol  3. Tobacco product like cigarette  4. Others |  |
| 203 | How often do you chew *khat?* | 1. Daily  2. More than one day per week  3. Once per week  4. Less than once per week |  |
| 204 | During chewing how many times and how many grams/packs do you chew? | How many times per day______  How many grams____________ or how many packs per day_______________  How many hours spent per chewing _________ |  |
| 205 | Have you consumed any alcohol within the past 30 days? | 1. Yes 2. No |  |
| 206 | Type of alcohol use | 1.beer 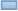 yes 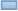 no  2.wine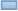 yes 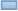 no  3.home made alcoholic drinks/specify |  |
| 207 | Do you currently smoke tobacco products | 1. Yes 2. No |  |
| 208 | How often do you smoke tobacco products*?* | 1. Daily  2. More than one day per week  3. Once per week  4. Less than once per week |  |
| Part III: Past and current o**bstetric related characteristics** | | | |
| 301 | How many months pregnant are you now? | ------------- |  |
| 302 | Have you planned this pregnancy? | 1. Yes 2. No |  |
| 303 | How many ANC visits did you have so far? | ________ |  |
| **Part IV: Medical history and current medicine/drug use status** | | | |
| 401 | Have you experienced diseases conditions prior to current pregnancy: | 1. Yes 2. No | If yes, specify (eg. Heart diseases, lung diseases, liver diseases, kidnney diseases, DM, HTN, HIV/AIDS, malaria etc) |
| 402 |  |  | 1.  2.  3. |
| 403 | Are you currently using medications? | 1. Yes 2. No | If yes, specify (eg. Antihypertensives, anti-diabetic, antipain, antibiotics etc.) |
| 404 |  |  | 1.  2.  3. |

**Pregnancy and fetal outcomes at delivery: participants ID**_____________________________________

| **Part V: Presentation and delivery outcomes** | | | |
| --- | --- | --- | --- |
| No | Variables | Responses | Remark |
| 501 | Indication for childbirth | 1. Spontaneous labor  2. Premature rupture of membranes  3. Fetal distress  4. Preeclampsia/gestational hypertension  5. Vaginal bleeding  6. Post-dates  7. Fetal growth restriction  8. Other, specify____________________ |  |
| 502 | Mode of Delivery | 1. Normal vaginal delivery  2. Instrumental (vacuum/forceps)  3. Planned Cesarean delivery  4. Emergency Cesarean delivery |  |
| 503 | Fetal presentation at delivery | 1. Cephalic  2. Transverse  3. Breech |  |
| 504 | Amniotic fluid at delivery | 1. Clear  2. Meconium-stained |  |
| 505 | Pregnancy outcomes | 1. Live birth  2. Still birth |  |
| 506 | Maternal status at the end of delivery | 1. Discharge  2. Referral  3. Admission  4. Death |  |
| 507 | If maternal death, what was underlying cause of death? | Specify, |  |
| 508 | Gestational age at birth | ________ Weeks |  |
| 509 | Birth weight | ___________grams |  |
| 510 | Are there noticed congenital anomalies at birth? | 1. Yes 2. No |  |
| 511 | If yes, types of congenital anomalies noticed: | 1. Neural tube defects  2. Microcephaly  3. Congenital malformations of ear  4. Suspected congenital heart defects  5. Orofacial clefts  6. Congenital malformations of digestive system  7. Congenital malformations of genital organs  8. Abdominal wall defects  9. Suspected chromosomal abnormalities  10. Reduction defects of upper and lower limbs  11. Talipes equinovarus/clubfoot  12. Others, specify |  |
