# Supplementary material for: The impact of chewing khat during pregnancy on selected pregnancy outcomes in eastern Ethiopia: A cohort study with a generalized structural equation modeling analysis approach
Source: PLoS One. 2024 Aug 9;19(8):e0308681. doi: 10.1371/journal.pone.0308681 (PMC11315279; doi:10.1371/journal.pone.0308681)
Supplement: Attachment — Submitted filename: Review Report.docx [file pone.0308681.s003.docx]

**Review Report**

The proposed study is interesting and the findings are good for the PloS one readers. However, there needs improvements before the acceptance of the manuscript. The authors are recommended to revise the manuscript according to the following instructions.

1- Add more literature related to the study and highlight the significance/novelty of the proposed study.
2- Discuss the statistical tool for the analysis of the dataset in detail.
3- Add abbreviations in a Table.
4- Highlight the findings, limitations, and merits of this study.
5- There are many grammar, spelling, and sentence mistakes, authors are directed to proofread the draft thoroughly.
6- Authors are suggested to cite some recent publications in the revised version especially, Abbas et al. (2023), and Abbas et al. (2024).

Abbas, Z., Nazir, H. Z., Riaz, M., Shi, J., and Abdisa, A. G. (2023). An unbiased function‐based Poisson adaptive EWMA control chart for monitoring range of shifts. *Quality and Reliability Engineering International*, *39*(6), 2185-2201. <https://doi.org/> <https://doi.org/10.1002/qre.3320>

Abbas, Z., Nazir, H. Z., Xiang, D., and Shi, J. (2024). Nonparametric adaptive cumulative sum charting scheme for monitoring process location. *Quality and Reliability Engineering International*, [*https://doi.org/10.1002/qre.3522*](https://doi.org/10.1002/qre.3522).
