# Supplementary material for: The impact of chewing khat during pregnancy on selected pregnancy outcomes in eastern Ethiopia: A cohort study with a generalized structural equation modeling analysis approach
Source: PLoS One. 2024 Aug 9;19(8):e0308681. doi: 10.1371/journal.pone.0308681 (PMC11315279; doi:10.1371/journal.pone.0308681)
Supplement: Attachment — Submitted filename: NOC_review_comments.docx [file pone.0308681.s004.docx]

General Comments

This paper examined the effect of chewing khat during pregnancy on a set of selected outcomes in Eastern Ethiopia. While there is good information within, the paper needs significant and extensive revisions if it is to be published. The completed paper should also be given to an editor who can help the authors improve the readability of the document as there are many errors and sentence-structure mistakes, which makes reading difficult. Overall, however there is valuable information within and if the writing and required revisions can be made, it is publishable.

Major issues

The authors title this paper a cohort study, but the study data was collected over the course of five months. In essence, this is a repeated measures survey.

Khat chewing was the exposure of interest, but the authors used self-report measures to assess this, rather than any validated or biological measure.

The authors state that the study follow-up period was antenatal care appointments, but don’t give any sense over what time period or the average time period such appointments last. For a person unfamiliar with the maternity care provided in Ethiopia, this is unclear.

The authors don’t say if there was a primary outcome and secondary outcomes they just mention pre-term birth and low birth weight, but how were these determined and why? Why not other outcomes? The abstract methods section does not mention any outcomes at all.

Was any blinding used in the assessment of the women in the study?

Did the study receive ethical approval? It isn’t stated in the manuscript.

There was a huge difference in the rate of attrition between the two exposure groups (double the number of khat chewers lost to follow up compared to non-khat chewers). This will have biased results to some degree, but it is not mentioned in the results or accounted for in the analysis, or even mentioned as a limitation.

In fact, the paper has no limitations section at all, despite there been many weaknesses. The most obvious weakness is the demographic and social differences between khat chewers and non-khat chewers. The two groups are profoundly different and while the authors try to account for the differences in their analysis, there could be substantial residual confounding explaining the findings.

Important characteristics of the women were not measured or reported, for example did the participants in the study give birth previously? Did they have any co-morbid health conditions that could also contribute to the outcome of preterm birth and low birth weight? Did participants smoke tobacco also? Are there any other potential biases in this study. The fact none are mentioned weakens this paper considerably.

Other issues

*Abstract*

Line 24: Change ‘less’ to ‘little is known’

Line 25: Drop the word ‘hence’ and start ‘The aim…’

Line 26: Remove the repetition of the tile and just say what the study design is.

Line 32-33: Name the primary and secondary outcomes

Line 45: Rewrite the conclusion section to be clearer and more precise.

*Introduction*

Line 63: Spelling ‘chat’ instead of ‘khat’.

Line 62 – 65: It isn’t clear from reading this what biological processes might make chewing khat during pregnancy dangerous. What is the mechanism and how might it adversely affect women and the two outcomes you are assessing?

*Methods and Materials*

Line 84: As above, it may not be accurate to call this a cohort study, but it is certainly not accurate to call it a longitudinal study.

Line 85: The study says it took place in a number of selected hospitals? How many sites?

Line 95: The study says, “Dire Dawa administration, Harari regional state and Jigjiga city were purposively selected due to exposure of interest’, but can further be said on this as it isn’t clear to the reader? The exposure is khat chewing, but what is it about these regions that relates specifically to khat chewing?

Line 97: Please re-write to clarify as it is not clear what “for the first or second time during the study period” means.

Other important questions remain about the methodology of the study. Were any inclusion or exclusion criteria used? Were any incentives given to participate? Is maternity care accessible in Ethiopia and do women pay for it? How many antenatal care appointments do women have and does it vary widely between women? It’s possible khat chewers (given their demographic differences) are less engaged with maternity services. It is also not stated in this study, but it is important to know, how many women were approached to participate and how many said no? Who recruited and consented the participants, were the women’s medics involved in recruitment? Was fully informed consent obtained?

Line 109: Change to “Measurement of the status to the exposure variable…”

Line 111: Rewrite the line beginning “The WHO…” to improve clarity, it isn’t well written at present.

Line 118: Rewrite the line beginning “Preterm birth…” as it is not clear.

General comment: Who collected the data? Were SFH measurements taken using case notes or by a medic or researcher?

*Results*

Table 1: Put the participant number totals in the column headings. There is no need to add the % sign in parentheses after each percentage as the ‘(%)’ in the column heading already denotes this to the reader.

Line 226: What was adjusted? It states what was adjusted for in the table, but it should also be stated in the text.

*Discussion*

Line 289: Use ‘two’ instead of the numeral ‘2’

Line 294: Change ‘during pregnancy cause labour induction, which may be preterm labour’ to ‘during pregnancy caused labour induction which may lead to preterm labour’

Line 298: Spell out acronym ‘PROM’ before use

Line 313: Change line to “This finding supports a case control…”
